# Occupational safety and health aspects of corporate social responsibility reporting in Japan: comparison between 2012 and 2020

**DOI:** 10.1186/s13104-022-06145-6

**Published:** 2022-07-23

**Authors:** Takahiro Shimizu, Tomohisa Nagata, Ayumi Fujimoto, Shunsuke Inoue, Masako Nagata, Koji Mori

**Affiliations:** 1 Department of Occupational Health Practice and Management, Institute of Industrial Ecological Sciences, University of Occupational and Environmental Health, Japan, 1-1 Iseigaoka, Yahatanishi-ku, Kitakyushu, Fukuoka 807-8555 Japan; 2 Department of Occupational Medicine, School of Medicine, University of Occupational and Environmental Health, Japan, Kitakyushu, Fukuoka Japan

**Keywords:** Reporting, Corporate social responsibility, Integrated report, Occupational safety and health, Environmental social governance, Industry category, Company size

## Abstract

**Objective:**

To survey the content of occupational safety and health (OSH) disclosed in corporate social responsibility (CSR)-related reports and integrated reports in 2020 and to compare the changes between 2012 and 2020 according to industry and company size.

**Results:**

Among all companies, 441 (20.3%) issued CSR-related reports and 590 (28.1%) issued integrated reports. The number (percentage) of companies that issued either CSR-related reports or integrated reports was 880 (40.5%). The percentages of both CSR-related reports and integrated reports increased with increased company size. The number (percentage) of companies reporting OSH in CSR-related reports was 391 (88.7%) and that in integrated reports was 493 (83.6%). The percentage of OSH reporting in CSR-related reports and integrated reports was high in secondary industries and low in tertiary industries.

**Supplementary Information:**

The online version contains supplementary material available at 10.1186/s13104-022-06145-6.

## Introduction

Corporate social responsibility (CSR) has recently garnered attention and social interest. CSR is defined as a company’s voluntary commitment to social and environmental considerations in the course of its business activities and relationships with stakeholders (all interested parties in organizational activities) [1]. The International Organization for Standardization issued ISO26000 to standardize CSR in 2010 [2]. The Global Reporting Initiative (GRI) has established sustainability reporting guidelines regarding how to disclose information on CSR activities, which states that companies are accountable to their stakeholders [3]. A previous study pointed out that the number of companies in Japan issuing CSR-related reports had increased as of 2012 [4]. In 2006, the United Nations introduced the concept of environmental social governance (ESG) in the Principles for Responsible Investment [5, 6]. The financial crisis triggered by the collapse of Lehman Brothers in 2008 increased the importance of corporate governance and ESG became recognized [5, 7].

In addition to CSR-related reports, integrated reports (meaning integrated financial and non-financial reporting) have begun to appear. The International Integrated Reporting Framework was established in 2013 [8], and this framework defined integrated reports as “a concise communication about how an organization’s strategy, governance, performance and prospects, in the context of its external environment, lead to the creation, preservation, or erosion of value over the short, medium, and long term” [8]. Previous studies have revealed that the number of companies issuing integrated reports in Japan has increased [9, 10].

Occupational Safety and Health (OSH) was included as a labor practice in ISO26000 [2]. The GRI also mentions OSH in GRI403 of the guideline, which lists the specific OSH activities to be disclosed [3]. A previous study reported that an increasing number of companies describe OSH in their CSR-related reports [4]. However, since the emergence of new forms of disclosing information, such as integrated reports, to the best of our knowledge, the methods and descriptions of information disclosure regarding OSH as CSR activities have not been clarified.

We conducted a survey of listed companies in Japan to determine the proportions of CSR-related reports and integrated reports published in 2020, and the proportion of content in these reports addressing OSH. We examined changes in these description between 2012 and 2020. The purpose of this study was to clarify the trend and development of information disclosure on OSH activities in CSR-related reports and integrated reports in Japan.

## Main Text

### Methods

We surveyed all 2172 companies listed in the first section of the Tokyo Stock Exchange in 2020 to determine the percentage of reports issued and the content of reports published on each company's website (Table 1). The included companies were obtained from among those listed on the website of the First Section of the Tokyo Stock Exchange on August 31, 2020. We surveyed CSR-related reports and integrated reports independently. In cases where both types of report were issued, we tabulated each report separately. The content of the survey addressed whether a report was issued and whether OSH was described. The distinction between CSR-related reports and integrated reports was determined using the name of the report and location of the report on the website. CSR-related reports can be found in the CSR activities, ESG activities, and sustainability sections of the website. In contrast, integrated reports are often posted in the investor relations section.

**Table 1:** Description of Japanese companies listed in the first section of the Tokyo Stock Exchange

	2012		2020	
	n	%	n	%
Total	1717		2172	
Industry category				
Fisheries, agriculture, and forestry	5	0.3	7	0.3
Mining	7	0.4	6	0.3
Construction	97	5.6	102	4.7
Manufacturing	844	49.2	914	42.1
Electricity and gas	17	1.0	22	1.0
Transportation, information, and communication	169	9.8	305	14.0
Commerece	300	17.5	381	17.5
Finance and insurance	129	7.5	139	6.4
Real estate	48	2.8	71	3.3
Services	101	5.9	225	10.4
Company size				
–49	70	4.1	139	6.4
50–299	274	16.0	462	21.3
300–999	594	34.6	756	34.8
1000–2999	501	29.2	511	23.5
3000–4999	133	7.7	126	5.8
5000–9999	69	4.0	80	3.7
10,000–	59	3.4	57	2.6
uncertain	17	1.0	41	1.9

*N* Number of companies

If the content of a report comprised only non-financial information, we determined it to be a CSR-related report. If financial information was presented together with non-financial information, we judged it to be an integrated report.

The inclusion criteria of a report were that it was published on a website in PDF format or in book form, that the report consisted of six or more pages, excluding the front and back covers, and that the report was written in Japanese. The exclusion criteria for reports were those prepared in English or languages other than Japanese, information disclosed only on the website, and no link to the report. We have additionally checked whether reports written in English have been issued.

We assessed the presence or absence of descriptions of OSH activities in each CSR-related report and integrated report. When there was at least one description related to safety or health in the workplace, we judged that there was a description of OSH activities; examples include items related to safety and health, such as efforts to prevent occupational accidents, mental health care, the existence of health education, and the establishment of safety and health committees.

An independent survey of 40 companies was conducted by four experts in occupational medicine and one expert in accounting, making up a total of five researchers [4]. The survey methods were based on those used in previous studies [4]. In the case of differences in the survey results, the causes of the differences were clearly defined, and the survey methods were summarized in a manual. From July 2020 to February 2021, a total of 20 people, 16 experts in occupational medicine and four medical students studying occupational medicine, conducted a web-based survey on the existence of CSR-related reports, integrated reports, and descriptions of OSH. We were unable to examine the content of all integrated reports during this period. Therefore, from July to September 2021, five medical students conducting research in occupational medicine carried out an additional survey of the integrated reports. In this case, we surveyed reports published in 2020 and excluded those published in 2021. Each researcher surveyed the same companies. In cases where the results of the survey were not matched (for example, the presence or absence of reports, categorized types of report, and OSH mentioned or not), we examined the causes and made modifications to standardize the survey.

### Statistical analysis

We classified the companies into 10 industries, as in a previous study, to determine the percentage of reports issued by industry and company size and the proportion of OSH. We categorized company size according to the number of employees. The 10 industries were Fisheries, agriculture, and forestry; Mining; Construction; Manufacturing; Electricity and gas; Transportation, information, and communication; Commerce; Finance and insurance; Real estate; and Services. The number of employees was classified into ranges of 49 or fewer, 50–299, 300–999, 1000–2999, 3000–4999, 5000–9999, and 10,000 or more; the percentage of reports issued was investigated.

We analyzed the data for companies that were listed in both 2012 and 2020 in order to determine reporting trends.

Statistical analysis was performed using Stata/SE 16 software for Windows (Stata Corp LLC, College Station, TX, USA).

## Results

Table 2 describes CSR-related and integrated reports issued in 2020. The number (percentage) of companies that issued CSR-related reports was 441 (20.3%), and integrated reports were issued by 590 companies (28.1%); the proportion of integrated reports was higher than that of CSR-related reports. In total, 880 companies (40.5%) issued CSR-related reports or integrated reports. The percentage of published CSR-related reports and integrated reports increased consistently with increased company size in 2020.

**Table 2:** Companies listed in first section of Tokyo Stock Exchange with published CSR372 related and integrated reports

	2012: CSRreports*		2020: CSRreports		2020: Integrated reports		2020: CSR reports orintegrated reports	
	n	%	n	%	n	%	n	%
Total	663	38.6%	441	20.3%	590	28.1%	880	40.5%
Industry category								
Fisheries, agriculture, and forestry	3	60.0%	2	28.6%	1	14.3%	3	42.9%
Mining	3	42.9%	2	33.3%	2	33.3%	3	50.0%
Construction	55	56.7%	37	36.3%	22	24.2%	54	52.9%
Manufacturing	427	50.6%	282	30.9%	309	35.6%	495	54.2%
Electricity and gas	14	82.4%	6	27.3%	14	66.7%	16	72.7%
Transportation, information, and communication	43	25.4%	33	10.8%	52	17.4%	77	25.2%
Commerece	66	22.0%	39	10.2%	67	17.8%	94	24.7%
Finance and insurance	29	22.5%	15	10.8%	80	58.4%	81	58.3%
Real estate	9	18.8%	10	14.1%	12	17.4%	16	22.5%
Services	14	13.9%	15	6.7%	31	13.8%	41	18.2%
Company size								
–49	15	21.4%	12	8.6%	18	13.0%	29	20.9%
50–299	51	18.6%	47	10.2%	56	12.5%	93	20.1%
300–999	152	25.6%	122	16.1%	133	18.2%	234	31.0%
1000–2999	241	48.1%	145	28.4%	184	37.9%	287	56.2%
3000–4999	91	68.4%	43	34.1%	84	70.0%	99	78.6%
5000–9999	50	72.5%	31	38.8%	56	72.7%	64	80.0%
10000–	52	88.1%	32	56.1%	43	76.8%	53	93.0%
uncertain	11	64.7%	9	22.0%	16	39.0%	21	51.2%

*n* number of companies, *CSR* corporate social responsibility

^*^Nagata et al. BMC Public Health (2017)

Table 3 provides descriptions of OSH activities in CSR-related reports and integrated reports in 2020. The percentage of OSH descriptions was 88.7% in CSR-related reports and 83.6% in integrated reports in 2020. By industry, the percentage of OSH activities described in CSR-related reports was more than 88.7% in the Electricity and gas, Fisheries, agriculture, forestry, Construction, Manufacturing, and Real estate sectors. The percentage of OSH activities described in integrated reports was more than 83.6% in Fisheries, agriculture and forestry, Mining, Construction, Electricity and gas, and Manufacturing.

**Table 3:** Companies describing occupational safety and health activities in CSR-related 383 or integrated reports

	2012: CSR reports*		2020: CSR reports		2020: Integrated reports	
	n(OSH)	%*1	n(OSH)	%*1	n(OSH)	%*2
Total	507	76.5%	391	88.7%	493	83.6%
Industry category						
Fisheries, agriculture, and forestry	2	66.7%	2	100.0%	1	100.0%
Mining	3	100.0%	1	50.0%	2	100.0%
Construction	47	85.5%	35	94.6%	22	100.0%
Manufacturing	337	78.9%	258	91.5%	279	90.3%
Electricity and gas	12	85.7%	6	100.0%	14	100.0%
Transportation, information, and communication	37	86.0%	27	81.8%	43	82.7%
Commerece	42	63.6%	27	69.2%	49	73.1%
Finance and insurance	14	48.3%	13	86.7%	53	66.3%
Real estate	7	77.8%	9	90.0%	10	83.3%
Services	6	42.9%	13	86.7%	20	64.5%
Company size						
–49	12	80.0%	7	58.3%	12	66.7%
50–299	40	78.4%	37	78.7%	46	82.1%
300–999	101	66.4%	109	89.3%	99	74.4%
1000–2999	182	75.5%	128	88.3%	156	84.8%
3000–4999	74	81.3%	39	90.7%	76	90.5%
5000–9999	42	84.0%	31	100.0%	53	94.6%
10000–	47	90.4%	31	96.9%	37	86.0%
uncertain	9	81.8%	9	100.0%	14	87.5%

*n* (OSH): number of companies describing OSH in reports, *CSR* corporate social responsibility, *OSH* occupational safety and health

^*^Nagata et al. BMC Public Health (2017)

^*^1: proportion of companies describing occupational safety and health activities among companies that published CSR-related reports

^*^2: proportion of companies describing occupational safety and health activities among companies that published integrated reports

With regard to company size, the percentage of OSH activities described in CSR-related reports was 58.3% in company with less than 50 employees, while more than 80% in companies with 300 employees and more. The percentage of OSH activities described in integrated reports was 66.7% in company with less than 50 employees, while more than 80% in companies with 1000 employees and more. Additional file 2: Figure S1 showed the percentage of OSH activities described in CSR-related or integrated reports in 2012 and 2020. For all company sizes, the percentage of OSH listed was higher in 2020 than in 2012.

Shows Additional file 1: Table S1 the number and percentage of companies that published English versions of their reports, by industry and size. The larger the employee size, the higher the percentage.

There were 1529 companies that were listed in both 2012 and 2020. In 1529, 604 companies (39.5%) had published CSR-related reports in 2012. Among these 604 companies, 154 companies (25.5%) had issued both reports in 2020, 156 (25.8%) companies had continued to issue only CSR-related reports, and 240 (39.7%) companies had transitioned from CSR-related reports to integrated reports.

## Discussion

In comparison with a previous study in 2012, we found that the number and percentage of reports issued by Japanese companies had changed. Additionally, we found that the publication of reports and description of OSH were unevenly distributed among industries and companies of different sizes. We discuss these factors and future perspectives below.

The percentage of CSR-related reports was lower than this percentage in 2012. We considered integrated reports to be similar to CSR-related reports, which deal with non-financial information, and we assessed whether any of these two types of report had been issued by the included companies [8]. The percentage of any of these reports issued increased slightly, indicating that the rising trend of issuing reports is ongoing.

The percentage of CSR-related reports and integrated reports issued increased with the size of the company. The ESG score provided by rating agencies, used to evaluate the ESG management of a company, has been reported to positively correlate with company size [11]. Larger companies are more active in ESG and may disclose a higher percentage of non-financial information and have a higher ESG score.

The percentage of OSH content in CSR-related reports increased in 2020, and the percentage of OSH in integrated reports was also high. However, the percentage of OSH was low for integrated reports in the tertiary sector. A program exists to survey and certify the health promotion efforts of employees in Japan, but the participation rate of tertiary industries in this health management survey is low [12]. It is considered that tertiary industries may have low awareness about OSH. OSH includes the element of safety, so it is possible that tertiary industries, which do not directly perform hazardous work, do not have sufficient descriptions about safety, yielding low results.

The percentage of OSH in CSR-related reports and integrated reports in 2020 increased with increased company size. In CSR-related reports and integrated reports in 2020, the proportion of OSH descriptions was lowest for companies with 49 or fewer employees. According to Japanese law, the health and safety system changes depending on whether there are 50 or more employees, [13], which may have an effect on the rate of reporting on health and safety among smaller companies.

In this survey, factors influencing the proportion of OSH content could not be clarified. However, the frequency of OSH reporting is higher in tertiary industries compared with secondary industries, according to Japanese statistics regarding industrial accidents [14]. Companies in the tertiary industry sector and other industries with low OSH reporting may be able to contribute to improving OSH for workers by promoting OSH disclosure.

This study had at least three strengths. First, we conducted a survey of all listed Japanese companies in 2020. Second, some studies have investigated changes in the number of integrated reports issued in Japan; however, to our best knowledge, none have investigated the changes in CSR-related reports and integrated reports. Third, to our best knowledge, no studies have assessed the rate of OSH in CSR-related reports and integrated reports. From the results of our study, it is possible to observe changes in the nature of non-financial information disclosure in Japan and the extent to which OSH is given attention in such disclosure.

## Limitations

This study had at least three limitations. First, variation among surveyors cannot be completely avoided. We tried to eliminate differences in understanding and perception among researchers by developing a manual based on past survey methods, involving all researchers in the conduct of surveys within the same company, and examining and defining differences in the results. Second, 75 integrated reports were written only in English, which were excluded from the survey. Therefore, the publication rate of reports and rate of OSH disclosure may vary from the figures in this survey. However, the number of integrated reports issued by listed companies in 2012 was very small (57 companies); we consider that this did not have a large impact on the results of the 2012 survey. Additionally, the number of reports in English was small compared with the total number of reports, and the analysis was conducted after excluding the number of integrated reports "issued", so we believe that the impact is small. Third, we were unable to identify the detailed characteristics of English versions of the reports because we did not review the content of these reports. In the case of more publications in English, a content survey of English versions would be desirable.

## Supplementary Information

**Additional file 1: Table S1.** Description of Japanese companies publishing reports written in English in 2020.

**Additional file 2: Figure S1.** Percentage of reporting occupational health and safety activities in CSR-related or integrated reports

## Data Availability

Data supporting these findings are stored by Occupational Health Practice and Management, Institute of Industrial Ecological Sciences, University of Occupational and Environmental Health, Japan. Requests for information should be directed to the corresponding author.

## Abbreviations

CSR: Corporate social responsibility
ESG: Environmental social governance
OSH: Occupational safety and health
GRI: Global Reporting Initiative
