# Supplementary figures and images for: Occupational safety and health aspects of corporate social responsibility reporting in Japan: comparison between 2012 and 2020
Source: BMC Res Notes. 2022 Jul 23;15:260. doi: 10.1186/s13104-022-06145-6 (PMC9308243; doi:10.1186/s13104-022-06145-6)

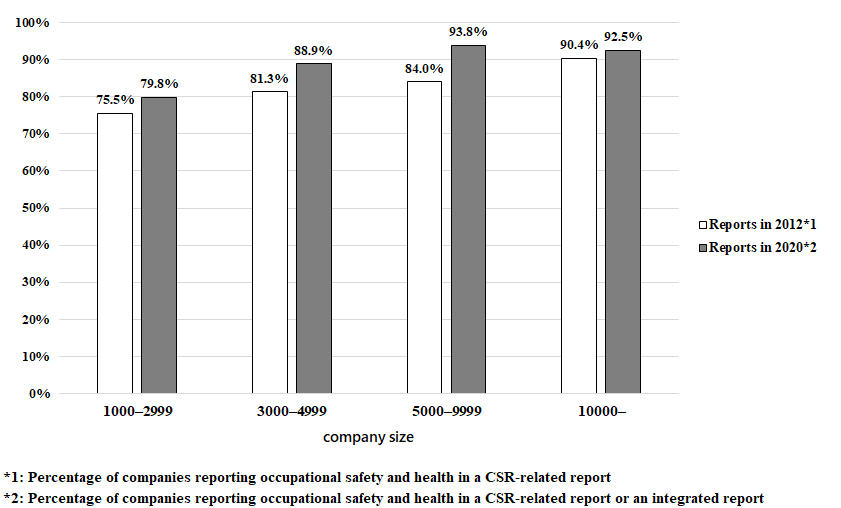

Supplement: Supplementary file 2 — Additional file 2: Figure S1. Percentage of reporting occupational health and safety activities in CSR-related or integrated reports [file 13104_2022_6145_MOESM2_ESM.bmp]
